# Exogenous OCT4 and SOX2 Contribution to In Vitro Reprogramming in Cattle

**DOI:** 10.3390/biomedicines11092577

**Published:** 2023-09-19

**Authors:** Lucas Simões Machado, Camila Martins Borges, Marina Amaro de Lima, Juliano Rodrigues Sangalli, Jacinthe Therrien, Laís Vicari de Figueiredo Pessôa, Paulo Fantinato Neto, Felipe Perecin, Lawrence Charles Smith, Flavio Vieira Meirelles, Fabiana Fernandes Bressan

**Affiliations:** 1 Post-Graduate Program of Anatomy of Domestic and Wild Animals, Faculty of Veterinary Medicine and Animal Sciences, University of São Paulo, São Paulo 05508-270, SP, Brazil; lucas.machado@unifesp.br (L.S.M.); camila.martins.borges@usp.br (C.M.B.); marina.amaro.lima@usp.br (M.A.d.L.); lawrence.c.smith@umontreal.ca (L.C.S.); meirellf@usp.br (F.V.M.); 2 Department of Veterinary Medicine, Faculty of Animal Sciences and Food Engineering, University of São Paulo, Pirassununga 13635-900, SP, Brazil; julianors@usp.br (J.R.S.); laisvpessoa@usp.br (L.V.d.F.P.); fantinato@usp.br (P.F.N.); fperecin@usp.br (F.P.); 3 Centre de Recherche en Reproduction et Fertilité, Faculté de Médecine Vétérinaire, Université de Montréal, Saint-Hyacinthe, QC J2S 7C6, Canada; jacinthe.therrien@umontreal.ca

**Keywords:** bovine, epigenetics, pluripotency, cellular reprogramming, OCT4, SOX2

## Abstract

Mechanisms of cell reprogramming by pluripotency-related transcription factors or nuclear transfer seem to be mediated by similar pathways, and the study of the contribution of OCT4 and SOX2 in both processes may help elucidate the mechanisms responsible for pluripotency. Bovine fibroblasts expressing exogenous *OCT4* or *SOX2*, or both, were analyzed regarding the expression of pluripotency factors and imprinted genes *H19* and *IGF2R*, and used for in vitro reprogramming. The expression of the *H19* gene was increased in the control sorted group, and putative iPSC-like cells were obtained when cells were not submitted to cell sorting. When sorted cells expressing OCT4, SOX2, or none (control) were used as donor cells for somatic cell nuclear transfer, fusion rates were 60.0% vs. 64.95% and 70.53% vs. 67.24% for SOX2 vs. control and OCT4 vs. control groups, respectively; cleavage rates were 66.66% vs. 81.68% and 86.47% vs. 85.18%, respectively; blastocyst rates were 33.05% vs. 44.15% and 52.06% vs. 44.78%, respectively. These results show that the production of embryos by NT resulted in similar rates of in vitro developmental competence compared to control cells regardless of different profiles of pluripotency-related gene expression presented by donor cells; however, induced reprogramming was compromised after cell sorting.

## 1. Introduction

Assisted reproductive biotechniques (ARTs) such as in vitro embryo production (IVP), intracytoplasmic sperm injection (ICSI), and mechanisms of in vitro induced reprogramming by either nuclear transfer (NT) or exogenous expression of pluripotency-related transcription factors (iPSCs generation) have important applications in regenerative medicine, and they may also greatly contribute to enhance animal production. In particular, in vitro reprogramming is a promising tool to overcome challenges in acquired infertilities or conservation of endangered species, and they may also lead to a better understanding of the underlying mechanism involved in initial embryonic development [1,2].

Nonetheless, ARTs are often performed in an environment that differs from the “in vivo” conditions, concerning, for example, the gaseous atmosphere and the nutrient supply in the culture. It has been shown that in vitro manipulations of gametes and embryos at the beginning of an organism’s development may lead to changes in epigenetic regulation, particularly due to the possible disruption of the gene expression pattern during the reprogramming cycles [3,4,5,6,7,8], which can lead to the occurrence of abnormalities in the development and even after birth of individuals derived from these techniques [3,4,5,6]. Indeed, a high incidence of epigenetic syndromes has been reported more frequently in ART-derived offspring than when natural reproduction occurs, particularly due to an abnormal epigenetic reprogramming leading to altered gene expression and dysfunctions in embryonic development and in the embryonic annexes in imprinted genes after in vitro reprogramming [7,9].

Several human epigenetic syndromes have been associated with disrupted imprinted genes, including Beckwith–Wiedemann or BWS [10], Silver–Russel or SRS [11], Angelman [12]**,** and Prader–Willi [13] syndromes. In particular, BWS and SRS are reported to be closely related to the *H19* and *IGF2* imprinted status. Usually, patients affected with syndromes resulting from disorders in the *H19/IGF2* locus present growth disorders, body asymmetry, intellectual disability, and the appearance of tumors [14]. A common condition in ruminants derived from ARTs is large offspring syndrome or LOS), with causes and phenotypes very similar to the BWS in humans [15,16,17].

Mechanisms of pluripotency acquaintance, in vivo or in vitro, seem to be mediated by the same pathways, eliciting nuclear remodeling and modulating gene expression. Two transcription factors, OCT4 and NANOG, were the first to be identified as essential for early embryonic development and for maintaining stem-cell pluripotency [18,19]. It was also shown that SOX2, another transcription factor, heterodimerizes with OCT4, regulating several genes in pluripotent cells [18,19,20,21,22]. Hence, not only are these transcription factors bound to their target DNA sites, the proteins are known to interact with each other and with chromatin remodeling agents, modulating the chromatin conformation and, therefore, the gene expression [23,24]. Interestingly, more recently, both OCT4 and SOX2 factors have been reported to have a considerable influence on the regulation of some imprinted genes, especially at locus *H19/IGF2*, known to be essential for normal embryo and placenta development [25,26,27].

In mouse embryos, Zimmerman et al. reported that the binding of OCT/SOX pluripotency factors to the *H19/IGF2* locus ICR contributed to hypomethylation in post-compaction embryos, thus relating the methylation status of these genes to the main factors of pluripotency [28]. Habib et al. (2014), through a study with 57 patients with BWS, demonstrated that some patients who present methylation gain in the *H19/IGF2* locus also present mutations in the binding site of the OCT4/SOX2 factors, showing that the SOX/OCT motifs within *H19/IGF2* ICR also participate in maintaining hypomethylation of the maternal allele [29].

It is, therefore, important to investigate possible factors involved in the induction and regulation of pluripotency acquisition, imprinting maintenance, and gene expression of the genes relevant to the development in cattle, possibly one of the livestock species where in vitro technologies are currently used in favor of animal production. Herein, we present an experimental in vitro model where pluripotency factors were studied together or separately regarding their influence on cellular genomic imprinting regulation and pluripotency acquisition in vitro.

## 2. Materials and Methods

All procedures were performed in accordance with the Guide for the Care and Use of Laboratory Animals of the National Institutes of Health and The ARRIVE Guidelines, as well as with the rules issued by the National Council for Control of Animal Experimentation (CONCEA, Ministry of Science, Technology, and Innovations and Communications, and in accordance with Law 11.794 of 8 October 2008, Decree 6899 of 15 July 2009) and in accordance with the provisions of the Resolution 466/12 of the National Health Council. Protocols were then approved by the Ethics Committee on Animal Use of the School of Veterinary Medicine and Animal Science University of São Paulo, Brazil (protocol number 8077020516) and by the Ethics Committee on the Use of Animals of the Faculty of Animal Science and Food Engineering, University of São Paulo, Brazil (protocol number 3526250717).

### 2.1. Bovine Fetal Fibroblast (bFF) Isolation and Experimental Design

The cell lines were obtained from three *Bos indicus* (maternal) × *Bos taurus* (paternal) fetuses at approximately 50 days of gestational age conceived after artificial insemination. The crossbred (F1) model was used to study allele-specific imprinted genes expression as previously described by our group and others, detailed below. After the removal of the head and organs, tissue was washed with PBS (phosphate-buffered saline) and minced into small fragments, followed by a 3 h incubation in collagenase IV (0.040 g/mL, Sigma-Aldrich Corp., St. Louis, MO, USA) at 38.5 °C. Next, the dissociated tissue was plated and cells were cultured in Iscove’s modified Dulbecco’s medium (IMDM, Thermo Fisher Scientific; Carlsbad, CA, USA) supplemented with 10% fetal bovine serum (FBS, Hyclone) and antibiotics (penicillin/streptomycin, Thermo Fisher Scientific; Carlsbad, CA, USA) [30]. All lineages were cryopreserved at low passages (p2–3) and thawed for experiments.

All three bovine fetal fibroblast (bFF) lineages were used for exogenous expression of OCT4, SOX2, or both, and then submitted to fluorescence analysis and cellular sorting. A previously validated bicistronic vector system for iPSC induction that allows for simultaneous real-time tracking of expression of the individual transgenes in single cells was used [31].

After cell recovery, bFF1 (male) was characterized regarding epigenetic maintenance at the *H19/IGF2* imprinted locus, and further reprogrammed by nuclear transfer (cells expressing OCT4, SOX2, and control) or induced reprogramming (cells named non-sorted control, sorted control, OCT4+, SOX2+, and OCT4 + SOX2).

**Figure 1:**
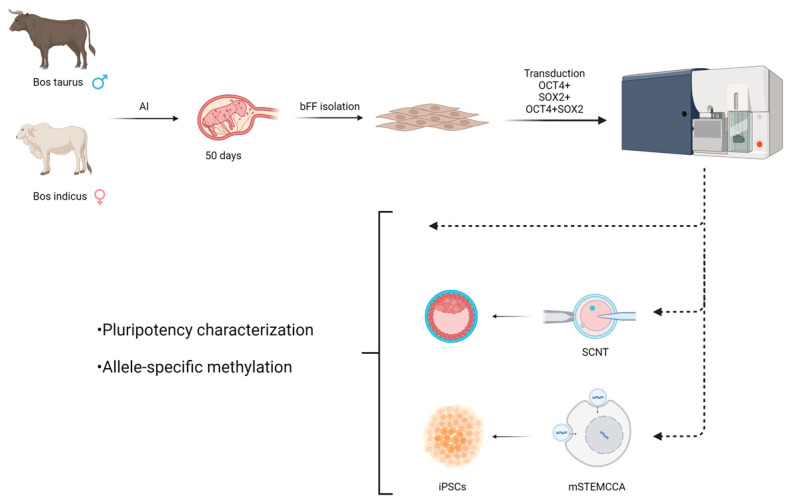
Experimental design showing the cell isolation from *Bos taurus × Bos indicus* animals, transduction, sorting, and cellular reprogramming through nuclear transfer or induced in vitro reprogramming.

The experimental design is briefly shown in Figure 1.

### 2.2. Generation of Fibroblasts Expressing Exogenous OCT4 and SOX2

For the production of the cells with exogenous expression of OCT4 and SOX2 and the association of both OCT4 and SOX2, the pLM-vexGFP-Oct4 and pLM-mCitrine-Sox2 vectors were used for lentivirus production as previously described [31]. The first contained human OCT4 (hOCT4) and a fluorescent reporter protein coding sequence for the vexGFP (excitable at 407 nm and emission at 535 nm, Addgene #22240); the second contained hSOX2 and the mCitrine (excitable at 516 nm and emission at 529 nm, Addgene #23242) fluorescent reporter. The use of bicistronic lentiviral vectors encoding the reprogramming factors being co-expressed with discernable fluorescent proteins guarantees the monitoring of expression of each individual reprogramming factor in cells during the course of reprogramming, in a stoichiometric and temporal manner.

Lentiviral particles of OCT4-vexGFP and SOX2-mCitrine were produced by lipofection of 293FT cells (Thermo Fisher Scientific; Carlsbad, CA, USA) with Lipofectamine 2000 (Thermo Fisher Scientific; Carlsbad, CA, USA), using 5 μg of pLM-vexGFP-Oct4 and pLM-mCitrine-Sox2 vectors, 1.2 μg of PLP1 and PLP2 and 2.4 μg of PLP/VSVG (ViraPower kit, Thermo Fisher Scientific; Carlsbad, CA, USA), following the manufacturer’s protocol. The supernatant (culture medium) was collected and refreshed at 48 h and 72 h after transfection, filtered, and used for transduction. Pluripotency was induced as previously described, using mouse OCT4, SOX2, c-MYC, and KLF4 transcription factors (mOSKM, mSTEMCCA) [32,33].

### 2.3. Flow Cytometry Analysis

After 72 h of transduction, protein expression was analyzed by flow cytometry. The gating strategy comprised using non-transduced cells as controls. Positive cells were sorted (BD FACSDiva software and BD FACSAria II SORP equipment—excitation laser 405 nm and detection filter 510/30 for the vexGFP protein and excitation laser 488 nm and 530/30 detection filter for the mCitrine protein). The recovered cells were re-cultured and induced to pluripotency; some were cryopreserved (experimental group pre-induction), and others were used in the subsequent analyses. On the basis of a higher transduction efficiency detected by flow cytometry, one cellular lineage was used for in vitro reprogramming.

### 2.4. Gene Expression of Imprinted and Pluripotency Genes

RNA was extracted with the RNeasy mini kit (Qiagen, Hilden, Germany) according to the manufacturer’s recommendations, and its quality and quantity were assessed by spectrophotometer (Nanodrop 2000). cDNA was synthesized with the High-Capacity cDNA reverse transcription kit (Thermo Fisher Scientific; Carlsbad, CA, USA) according to the manufacturer’s recommendations. Experimental groups were quantified regarding their transcripts of imprinted genes *H19* and *IGF2R* and genes related to pluripotency OCT4 and SOX2. Beta-actin (ACTB) and CCR4-NOT transcription complex subunit 11 (ACTB and C2ORF29 or CNOT11) were used as housekeeping genes.

Relative analysis of transcripts was performed by RT-qPCR (7500 Fast Real-Time PCR System, Thermo Fisher Scientific; Carlsbad, CA, USA) using a commercial assay in duplicate (Power SYBR^®^Green PCR Master Mix, Thermo Fisher Scientific; Carlsbad, CA, USA), where 5 µL of the sample cDNA was added to a 20 µL final volume reaction. The primers’ (Table 1) final concentration was 200 nM, and standard curves were performed to evaluate the efficiency of each gene. The qPCR reaction consisted of a denaturation step of 95 °C for 5 s, and an annealing temperature of 60 °C, for 40 cycles. Data were analyzed using the delta–delta CT method [34].

### 2.5. Allele-Specific Methylation Analyses of the DMR at the H19/IGF2 Locus

DNA extraction was performed with the DNeasy Blood and Tissue Kit (Qiagen, Hilden, Germany) according to the manufacturer’s recommendations, and quality and quantity were determined by a spectrophotometer (Nanodrop 2000). The EpiTect Bisulfite Kit (#59104 Qiagen, Hilden, Germany) was used according to the manufacturer’s recommendations in a thermocycler for 5 min at 99 °C, 25 min at 60 °C, then 5 more min at 99 °C, 85 min at 60 °C, back to 5 min at 99 °C, 175 min at 60 °C, and finally, 20 °C overnight.

Amplification of fragments from the H19/IGF2 DMR (proximally −3327 to −2675 base pairs away from exon 1 was performed using the primers U-H19 F1 and U-H19 R4 (Table 1). The PCR reaction contained 38.5 µL of ultrapure H_2_Od, 5 µL of Buffer TPN 10× (Invitrogen), 1.5 µL of dNTP (Invitrogen), 1.5 µL of MgCl_2_, 1 µL of primer (one for each, forward and reverse), and 0.5 µL of Platinum Taq (Invitrogen, #10966) for each sample, before adding 1 µL of DNA. Each PCR reaction was performed in triplicates. The protocol used was 1 min of plate pre-heating, 50 cycles of 30 s at 94 °C, 30 s at 53 °C and 30 s at 72 °C, and one final 7 min step at 72 °C. Amplified samples were run in a 1.2% agarose gel alongside a 1 Kb ladder, and the master mix lacking DNA as control was purified from the agarose gel and sequenced.

Global and allelic expression analysis of imprinted genes was realized as described by Suzuki and collaborators [35]. A single-nucleotide polymorphism (SNP) at the IGF2/H19 locus between *Bos indicus* and *Bos taurus* allowed for allele-specific DNA methylation analysis after sequencing and allele-specific gene expression analysis. The nucleotide guanine at the sequence TTTAT**G**TATTA indicates *Bos indicus* origin; therefore, the allele is of maternal origin. If the nucleotide adenine were present in its place, that would indicate an allele of paternal origin.

### 2.6. In Vitro Induced Reprogramming into Pluripotency

Three repetitions (R1, R2, and R3) were submitted to the pluripotency induction. The lentiviral particles containing mouse OSKM (mSTEMCCA) were produced by lipofection of 293FT cells, as previously described [32,33]. At 5 or 6 days after transduction, the cells were transferred to culture plates containing a monolayer of mitotically inactivated (mitomycin C, M4287 Sigma-Aldrich) mouse embryonic fibroblasts (MEFs).

During the cellular reprogramming, the cells were cultured in iPSC medium consisting of DMEM/F12 Knockout (Thermo Fisher Scientific), supplemented with 20% knockout serum replacement (KSR, Thermo Fisher Scientific), 1% glutamine (Thermo Fisher Scientific), 3.85 μM β-mercaptoethanol (Thermo Fisher Scientific), 1% non-essential amino acids (Thermo Fisher Scientific), 10 ng/mL bFGF (Peprotech) and antibiotics (penicillin/streptomycin, Thermo Fisher Scientific). Morphologically typical colonies were manually picked at the first passage, and clonal lines were further dissociated for passaging with TrypLE Express (Life Technologies).

### 2.7. Somatic Cell Nuclear Transfer

Fetal fibroblasts expressing either OCT4-vexGFP or SOX2-mCitrine were analyzed for OCT4 and SOX2 gene expression through qPCR and flow cytometry analysis, which enabled the sorting of positive cells used as donor cells for somatic cell nuclear transfer procedures as previously described [36,37]. Briefly, bovine oocytes obtained from slaughterhouses were in vitro matured for 18 h, enucleated, and reconstructed with fibroblasts expressing OCT4-vexGFP (n = 182, in four replicates), SOX2-mCitrine (n = 203, in four replicates), or control cells (non-transduced, n = 178 and n = 149, in four replicates as control of OCT4 or SOX2 expressing cells each). After reconstruction, embryos were activated with ionomycin (5 μM, 5 min) and 6-DMAP (2 mM, 3 h) and in vitro cultured until blastocyst stage (7 days) in SOF medium supplemented with 2.5% FBS and 3mg/mL BSA.

### 2.8. Statistical Analysis

Data obtained from the experimental procedures were analyzed using the statistical program Statistical Analysis System (SAS University Edition), with previous verification of the normality of the residues by the Shapiro–Wilk test (PROC UNIVARIATE) and submitted to analysis of variance. Gene expression data were then submitted to the Bonferroni test. A significance level of 5% was considered for all statistical analyses.

## 3. Results

### 3.1. bFF Expressing Exogenous OCT4 and SOX2

**Figure 2:**
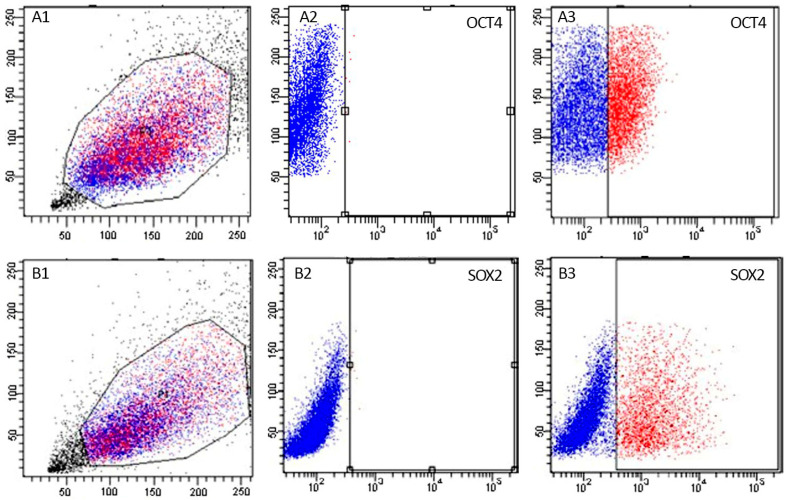
Representative scatter plots of flow cytometric analysis of stable lines expressing OCT4-vexGFP (**A1**–**A3**) and SOX2-mCitrine (**B1**–**B3**) used for sorting nuclear donor cells. Blue dots represent negative cells (non-fluorescent) and red dots present positive cells (fluorescent cells). In (**A1**,**B1**), the Y-axis represents side scatter (SSC) and X-axis represents forward scatter (FSC). (**A2**,**B2**) represent the control groups, where the Y-axis represents the cell count and X-axis represents the fluorescence in arbitrary units. (**A3**,**B3**) represent the hOCT4 and the hSOX2 groups, where the Y-axis represents the cell count and X-axis represents the fluorescence in arbitrary units.

The exogenous expression of OCT4 and SOX2 was confirmed by flow cytometry (Figure 2), where the positive populations were sorted out and recovered for in vitro culture and reprogramming procedures. The percentage of positive cells in each cell line and treatment group is presented in Table 2. The post-sorting purity percentage was 90% or greater.

Due to higher fluorescence detection of bFF1, the post-sorting recovery was more efficient, enabling its utilization for the subsequent experiments. Cells expressing both OCT4 and SOX2 presented very high proliferation levels, followed by early senescence. Such behavior was observed over three repetitions; therefore, they were used for induced pluripotency, and nuclear transfer was not conducted.

### 3.2. Quantitative Gene Expression Analyzes of Imprinted Genes or Genes Related to Pluripotency

The experimental groups from the three repetitions were induced to pluripotency (R1, R2, and R3) and evaluated regarding the expression of pluripotency factors OCT4 and SOX2 and imprinted genes H19 and IGF2R. OCT4+ cells showed higher OCT4 expression, and SOX2+ cells had greater SOX2 expression, as expected. This analysis enabled us to detect the high exogenous expression of the target genes.

**Figure 3:**
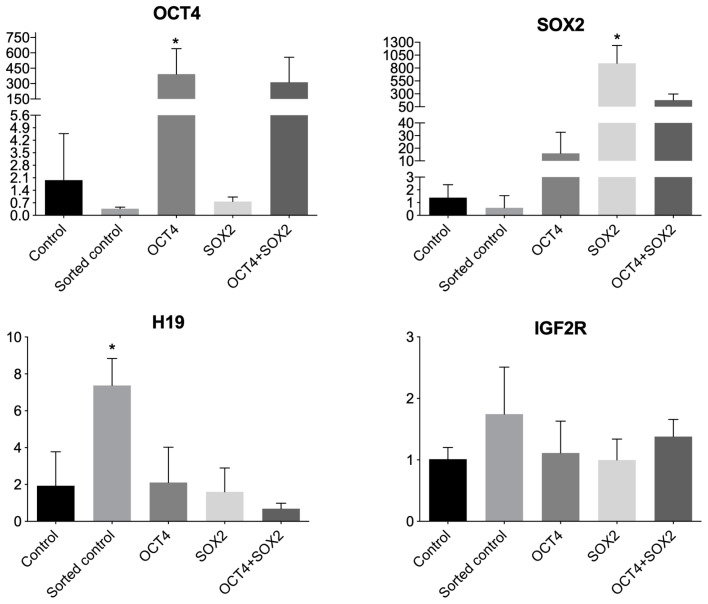
Quantitative gene expression of OCT4, SOX2 (pluripotency-related genes), H19, and IGF2R (imprinted genes) in bovine cells from experimental groups: control, sorted control, expressing exogenous OCT4, expressing exogenous SOX2, or expressing both (OCT4 + SOX2), in arbitrary units. The X-axis represents arbitrary units and the Y-axis represents the experimental groups. Bars presenting an asterisk (*) showed a statistical difference (*p* < 0.05).

Double-positive cells (OCT4+/SOX2+) OCT4 + SOX2 cells showed an increase in both OCT4 and SOX2 expression, albeit not statistically significant, probably because the expression was similar to groups expressing only OCT4 and SOX2, and there is apparently an interaction between exogenous OCT4 expression and endogenous SOX2 expression. It is noteworthy that SOX2+ cells had an approximately 10-fold OCT4 level increase when compared to the control (Figure 3).

The analysis of the imprinted gene H19 expression showed an increased expression in control sorted group, which was not expected. It is speculated herein that the pluripotency factors somehow protect the locus against possible deregulation caused by the sorting procedure, and such possibility must be further investigated with analyses of more repetitions and the methylation pattern of this specific locus.

No differences among the groups were observed when the imprinted gene IGF2R was analyzed; however, there was an approximately 50% increase in expression levels in the sorted control group when compared to the non-sorted control, indicating, once more, a possible effect caused by the flow cytometry analysis and sorting on the regulation of imprinted genes. More repetitions are needed to further understand this possibility.

**Figure 4:**
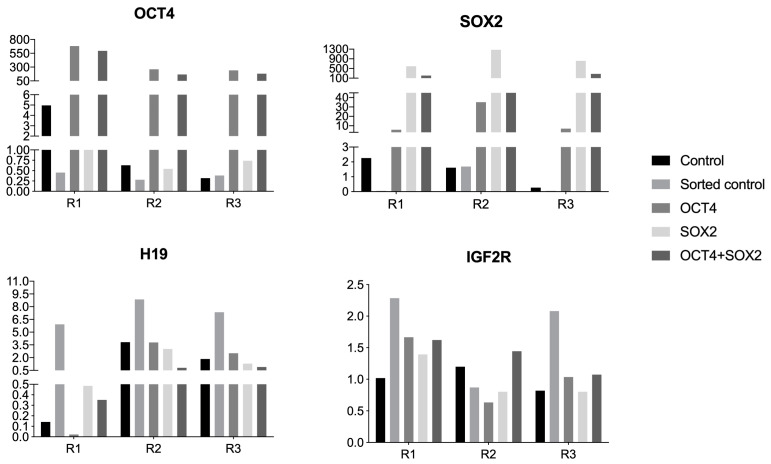
Quantitative gene expression of OCT4, SOX2 (pluripotency-related genes), H19, and IGF2R (imprinted genes) in bovine cells from experimental groups: control, sorted control, expressing exogenous OCT4, expressing exogenous SOX2, or expressing both (OCT4 + SOX2), in arbitrary units. R1, R2, and R3 are representative bars for each lineage (repetition).

The analysis of each repetition showed that, even though the gene expression pattern seemed very similar in the groups, the non-sorted R1 had a unique pattern (Figure 4). R1 had a higher expression of OCT4 and a lower expression of H19 in relation to R2 and R3, indicating a possible relationship between them, and R1 was the group able to produce iPSCs colonies more efficiently. The existence of more reprogrammable populations has already been described in the literature [38,39], and more studies are necessary to reveal if such pre-disposition may be related to the regulation between imprinted and pluripotency genes.

### 3.3. Allele-Specific Methylation Analyses of the DMR at the H19/IGF2 Locus

The methylation was analyzed, and the bisulfite conversion rate (number of non-converted cytosines in relation to all convertible cytosines) was considered appropriate when superior to 90% (Table 3).

The percentage of total methylation, as well as the methylation at the CTCF region, was calculated using the number of methylated and not methylated CpG islands. In general, the paternal allele (taurus) was methylated, and the maternal allele (indicus) was not methylated (Table 4).

### 3.4. Pluripotency Induction (iPSC Production)

bFF1 was used for pluripotency induction. Interestingly, non-sorted cells generated biPS colonies, whereas sorted cells (control non-transgenic, OCT4-, SOX2-, and OCT4- + SOX2-expressing cells) did not generate biPS cells.

The percentage of colonies formed is described in Table 5 (the number of colonies formed divided by the number of plated transduced cells). The groups transduced with hOSKM did not produce iPSC colonies.

**Figure 5:**
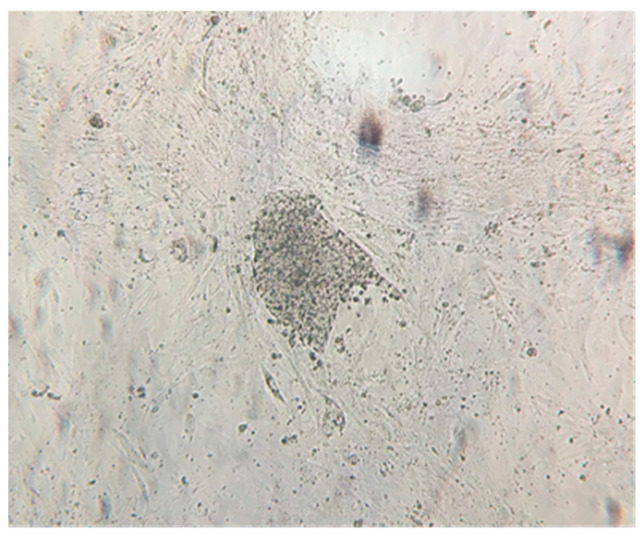
Representative image of a reprogrammed colony of cells from the non-sorted control before first picking (p0), 200×.

Cells showed typical colony morphology with approximately 15 to 20 days post transduction (Figure 5).

Colonies were manually picked at the first passage and later enzymatically passaged. Among the repetitions, different passages on bFF1 were used since the same cell line was cultured continuously while used on the repetitions. Therefore, R1 had the lowest number of passages in vitro.

### 3.5. Somatic Cell Nuclear Transfer Using hOCT4 and hSOX2 Overexpressing Donor Cells

**Figure 6:**
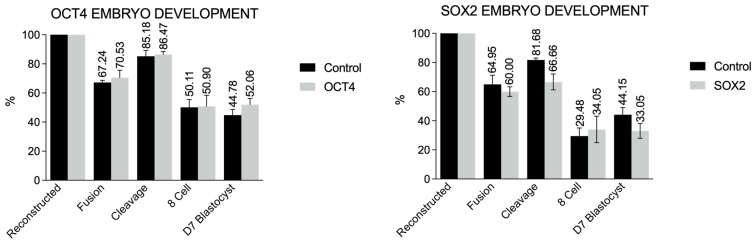
Development competence (percentage of reconstructed and fused embryos, cleavage, and development—8 cell and blastocyst rates) of nuclear transfer-derived embryos produced with donor cells expressing exogenous OCT4, SOX2, or none (control cells).

Fusion rates were 60.0% vs. 64.95% and 70.53% vs. 67.24% for SOX2 vs. control and OCT4 vs. control groups, respectively; cleavage rates (48 h after activation) were 66.66% vs. 81.68% and 86.47% vs. 85.18%, respectively; blastocyst rates (192 h after activation) were 33.05% vs. 44.15% and 52.06% vs. 44.78%, respectively (Figure 6).

There were no differences in the rate of fusion, cleavage, percentage of embryos in eight cells, and capacity of development to blastocysts on the seventh day of in vitro culture between clone embryos reconstructed with modified or not cells.

## 4. Discussion

During development, a mammal’s genome is epigenetically reprogrammed on two different and essential occasions: gametogenesis and early embryogenesis. The reprogramming processes occur in the primordial germ cells (PGCs), where epigenetic markers are erased, and new ones are established at specific moments, both before and after fertilization. After fertilization, a second wave of global demethylation occurs, except for imprinted genes, followed by de novo methylation, which sets a new epigenetic layout allowing totipotency and following cell line committed differentiation [40,41]. Epigenetic modifications may be inherited but also modified by the environment, thus explaining the more frequent and different phenotypical alterations observed in ART-generated individuals [42]. In this study, the creation of an in vitro experimental model that enables the study of OCT4 and SOX2 transcription factors, as well as their combination, in the regulation of the imprinting in the H19/IGF2 locus in bovine cells and cells reprogrammed in vitro was proposed.

In this study, bovine cell lines expressing pluripotency exogenous factors OCT4, SOX2, or both were produced. These lines are important to better understand the acquisition and maintenance of the pluripotency process in vitro. The sorting of positive cells for those factors allows us to use only those cells that have integrated the factors, increasing, in theory, reprogramming efficiency. The production of such factors was accomplished by the lentiviral approach, and the lentiviral production was confirmed through fluorescence analysis of the 293FT cells after transfection. After 5 or 6 days post transduction, the percentage of positive cells for the reporter genes was quantified by flow cytometry and used as a lentiviral transduction efficiency parameter. Such measurement is valid for the qualitative and quantitative analysis of the efficiency of integration of the transgene since the multiplicity of infection of these vectors results in a linear title of the average fluorescence intensity of each corresponding fluorescent protein [31]. The same group reported that a threefold increase of OCT4 in relation to the SOX2, KLF4, and c-MYC levels raised the reprogramming efficiency, and the opposite resulted in a drastic decrease in reprogramming efficiency [31]. In 2011, Yamaguchi and collaborators reported that decreased SOX2 levels increased efficiency in partially reprogrammed cells production [43], showing that both expression and interaction of OCT4 and SOX2 need to be finely regulated for acquiring and maintaining pluripotency in vitro.

The expression of the imprinted genes H19 and IGF2 was also analyzed on the cell lines produced, and the methylation of the DMR at the H19/IGF2 locus. Nevertheless, the hypothesis that the overexpression of OCT4 and SOX2 in bovine cell lines is not only possible but leads to modifications in expression and imprinting pattern in the H19/IGF2 locus, as well as in bovine reprogramming into pluripotency by TNCS or iPSC generation efficiency, was not confirmed in this study.

Even though further analyses are needed, it is possible to observe that the methylation pattern of the non-sorted group was slightly different from the others that were submitted to the same sorting process; the maternal allele was, herein, completely demethylated. To infer if the expression of the exogenous factors acts in a protective way toward the H19/IGF2 locus from external interferences, such as sorting or reprogramming, further analyses are still needed.

The generation of induced pluripotency models (induced pluripotent stem cells) made it possible to study the process of in vitro reprogramming more precisely. In this study, cell reprogramming after OSKM transduction was observed only in the control group, and the study of whether the imbalance between OCT4 and SOX2 expression may hamper induced reprogramming will be of great importance to better understand the role of these pluripotency factors in the acquisition and maintenance of the epigenetic patterns of reprogrammed cells.

Nonetheless, the results obtained herein (reprogrammed cells generated only from the non-sorted control group and, within that group, a higher number of colonies on the first repetition, fewer colonies on the second repetition, and none on the third), two effects can be inferred: (1) a sorting effect, and (2) an in vitro passage number effect. The environmental factor must also be considered, with possible effects caused by the laboratory routine itself as changes in the culture medium lot or supplements.

Moreover, the embryo production by NT from cells expressing hOCT4 or hSOX2 resulted in similar in vitro embryonic development rates regardless of the gene expression profiles of factors related to the pluripotency of the nucleus donor cells.

Lastly, in this study, we analyzed whether the overexpression of two important pluripotency-related genes could be related to the success of cellular reprogramming and to a specific imprinting deregulation, which is commonly reported (H19/IGF2 locus). There is limited research on the influence of specific genes in the in vitro reprogramming of animals other than rodents and primates. Herein, we showed that the overexpression of pluripotency-related factors, proven by a reporter gene and molecular analysis, is not able to completely impact the epigenetics and the efficiency of the in vitro reprogramming in cattle, and other strategies should be implemented in order to generate healthy cloned cattle or bona fide pluripotent stem cells in this species.

## 5. Conclusions

The results described in this study allow us to conclude that the production of cells expressing exogenous pluripotent factors was successful, as shown by the gene expression experiments. Cellular reprogramming to pluripotency by cloned embryo production was achieved in the present study when cells expressing OCT4 or SOX2 were used as donor cells; however, in our conditions, these cell lines did not result in iPSCs after induced reprogramming in vitro. There was interference from the flow cytometer analysis and sorting process in the expression of the imprinted gene H19 in at least one of the experimental groups.

The production of embryos by NT of hSOX2- or hOCT4-expressing donor cells resulted in similar rates of in vitro developmental competence compared to control cells regardless of different profiles of pluripotency-related gene expression presented by donor cells. A better understanding of the contribution of each reprogramming factor used in induced reprogramming will establish strategies to enhance in vitro reprogramming performance. Such knowledge will contribute to in vitro animal production by increasing the cloning efficiency at term and regenerative medicine through the derivation and adequate culture of reprogrammed pluripotent stem cells.

## Figures and Tables

**Table 1:** Primer sequences for the quantitative analysis of transcripts and methylation analysis at the IGF2/H19 locus.

Name	5′–3′ Sequence
ACTB_FWD	GCGGACAGGATGCAGAAA
ACTB_REV	ACGGAGTACTTGCGCTCAG
C2ORF29_FWD	ACTGAGCCTGACCATGCGATC
C2ORF29_REV	GGCTGGAGTGAGGCCAATATG
H19_FWD	AGTGGGAGGGGCATTGGACT
H19_REV	GACCATATCATATCCCTCTGTGC-
SOX2_FWD	ATGGGCTCGGTGGTGAAGT
SOX2_REV	TGGTAGTGCTGGGACATGTGA
OCT4_FWD	GCAAACGATCAAGCAGTGACTAC
OCT4_REV	GGCGCCAGAGGAGAGGATACG

**Table 2:** Percentage of fluorescent cells by flow cytometry in three bovine fetal fibroblast cell lines.

	hOCT4%	hSOX2%	hOCT4 + hSOX2%
bFF1	79.8	10.2	1.3
bFF2	22.7	4.2	0.4
bFF3	18.7	3.9	0.2
Average	40.4	6.1	0.63

**Table 3:** Bisulfite conversion efficiency rate.

	Non-Sorted Control	Sorted Control	OCT4+	SOX2+	OCT4 + SOX2
Conversion rate	98.03%	98.23%	95.09%	98.89%	98.32%

**Table 4:** Methylation percentage in the DMR at the H19/IGF2 locus in the experimental groups.

	Non-Sorted Control (%DMR; %CTCF)	Sorted Control (%DMR, %CTCF)	OCT4+ (%DMR, %CTCF)	SOX2+ (%DMR, %CTCF)	OCT4 + SOX2 (%DMR, %CTCF)
Maternal allele	21.42; 33.33	0; 0	21.24; 6.67	2.4; 5.55	5.95; 11.11
Paternal allele	100; 100	92.85; 100	100; 100	96.42; 100	92.3

**Table 5:** Percentage of iPSC colonies formed in each experimental group in all three repetitions (percentage and number of colonies).

	Non-Sorted Control	Sorted Control	OCT4+	SOX2+	OCT4 + SOX2
R1	0.00035 (7)	0	0	0	0
R2	0.00005 (1)	0	0	0	0
R3	0	0	0	0	0

## Data Availability

Data are contained within the article.
